# Modern Herbal Nanogels: Formulation, Delivery Methods, and Applications

**DOI:** 10.3390/gels8020097

**Published:** 2022-02-07

**Authors:** Rakesh K. Sindhu, Rubal Gupta, Gaurish Wadhera, Pradeep Kumar

**Affiliations:** 1 Chitkara College of Pharmacy, Chitkara University, Rajpura 140401, India; rubal19104.ccp@chitkara.edu.in (R.G.); gaurish19032.ccp@chitkara.edu.in (G.W.); 2 Wits Advanced Drug Delivery Platform Research Unit, Department of Pharmacy and Pharmacology, Faculty of Health Sciences, School of Therapeutic Sciences, University of the Witwatersrand, Johannesburg 2193, South Africa; pradeep.kumar@wits.ac.za

**Keywords:** nanogels, herbal bioactives, hydrogels, polysaccharides

## Abstract

This study examined the most recent advancements in nanogel production and drug delivery. Phytochemistry is a discipline of chemistry that studies herbal compounds. Herbal substances have aided in the development of innovative remedies for a wide range of illnesses. Several of these compounds are forbidden from being used in medications due to broad medical characteristics and pharmacokinetics. A variety of new technical approaches have been investigated to ameliorate herbal discoveries in the pharmaceutical sector. The article focuses on the historical data for herb-related nanogels that are used to treat a variety of disorders with great patient compliance, delivery rate, and efficacy. Stimulus-responsive nanogels such as temperature responsive and pH-responsive systems are also discussed. Nanogel formulations, which have been hailed as promising targets for drug delivery systems, have the ability to alter the profile of a drug, genotype, protein, peptide, oligosaccharide, or immunogenic substance, as well as its ability to cross biological barriers, biodistribution, and pharmacokinetics, improving efficacy, safety, and patient cooperation.

## 1. Introduction

Nanogels have emerged as suitable vehicle for delivering and releasing medications to patients in recent years as one of the many dimensions of nanomedicine—the junction of nanotechnology, medicine, and pharmaceuticals. Nanogels are crosslinked polymer networks that are nanoscale in size and capable of absorbing enormous amounts of water [1]. Nanogels are hydrogels with a size of nanometers or less. A hydrogel is a polymer-based gel that is made by connecting polymer chains to form a macromolecular network. Hydrogels can be made in a variety of ways [2], but all require the creation of polymeric monomers, which must then be polymerized with functional cross-linker molecules to form a ‘net-like’ polymer structure [2]. Pharmaceuticals can be loaded into the pores and then released later through the pores. Nanogels, on the other hand, are essentially hydrogels but on a 20–200 nm scale. Emulsion polymerization is used to make the vast majority of nanogels. Patients can be given nanogels orally, pulmonary, nasally, parentally, or intra-ocularly. The medications are released from the nanogels in a variety of ways, but the mechanism including activation by external stimulation alters internal properties. Due to this physical change, which causes the polymer network to swell or compress, the medicinal payload is delivered to the desired area [3,4].

Depending on the release mechanism used, this stimulation could come from the body’s immediate surroundings or an external stimulus source. A certain pH and a change in temperature within a specific volume are the most common internal–external components that produce a physical change (also known as the volume phase transition temperature). On the other hand, light is the most common external stimulation because it initiates photochemical and photo isomerization processes, allowing the drug or the drug carrier itself to be released [3,4].

Nanomedicines have shown promising results in improving the bioavailability of a variety of chemical and herbal bioactive compounds [5]. Nanogels, considered as a nanomedicinal product, offer exceptional stability, drug loading capacity, biologic consistency, strong penetration ability, and the ability to respond to environmental stimuli. Nanogels have been considered prominent in a variety of sectors, including gene delivery, chemotherapeutic medication delivery, diagnostics, organ targeting, and herbal medicines [4]. In recent years, nanogels have been used in biotechnology, majorly used in dealing with genetics, protein synthesis, and enzyme immobilization. They serve as an asset for revolutionary treatment systems in medicine [6]. Furthermore, a type of nanogel (amphiphilic) is also employed because of its high drug loading capacity obtained using aggregation and sedimentation [7]. New generation nanogels’ main objectives are to provide safer and effective drug target delivery and are even believed to show effective responses in tissue engineering [8].

## 2. Nanocarrier as a Drug Delivery Mechanism

A nanocarrier is a type of nanomaterial that is used to convey another chemical, such as a medicine. Archetypes such as micelles, polymeric systems, carbon-based materials, and liposomes are often utilized as nanocarriers [9]. Nanocarriers are now being researched for application in medication delivery, and their particular properties suggest that they could be useful in chemotherapy [10,11]. Nanomaterial can enter the body by a variety of routes, including the respiratory system, the skin, the digestive tract, and medication injection, before being transported to organs and may exert severe biological effects such as inflammatory responses, oxidative stress, and DNA damage. As NPs possess an ability to load and deliver an amazing spectrum of drugs to almost any organ of the body, delivering focused, regulated, as well as prolonged therapeutic effects, they have become an important subject of drug delivery studies [12] as shown in Table 1. Examples of nanogels are reported for drug delivery applications including both hydrophobic and hydrophilic agents, and even oligodeoxynucleotides [13].

## 3. Nanotechnological Approach

At the nanoscale, nanotechnology provides a unique perspective for analyzing and controlling numerous biological and medicinal processes. Medicine and biology will forever be changed because of it. Nanomedicines have the advantage of precisely targeting therapy to the target cells while leaving healthy cells unharmed. The influence of developing a nanotechnology-based drug delivery system on accuracy and efficacy is significant; consequently, creating a nano-drug with an accurately administered delivery rate [17].

### 3.1. Features of Nanogel

#### 3.1.1. Targeting Delivery

Nanogels carriers are frequently delivered at specific sites by binding to their surface due to its dependency and focusing on variables of their responsiveness to local factors, or by other “passive” techniques that involve retention inside the physiological spaces.

#### 3.1.2. Low Level of Toxicity

The nanogels must be biofriendly and non-toxic, as well as perishable with non-toxic breakdown products which can be eliminated swiftly out from body.

#### 3.1.3. Controlled and Sustained Medication Delivery

Drug delivery should occur at the target location, ensuring that each treatment is delivered efficiently with fewer side effects. To achieve therapeutic aims, drug loading should be high [18].

#### 3.1.4. High Encapsulation Stability

Drug molecules embedded in nanogels must not be transported out or leaked prematurely while delivering the most significant therapeutic benefit with the least amount of toxicity or side effects [19].

#### 3.1.5. Size Control

Physicochemical strategies are often employed to adjust nanogel size and surface features to minimize somatic cell clearance and change active cell targeting or the passive one. Nanogels ought to be small enough to be able to the pass through capillaries and the tissues via paracellular or transcellular pathways.

### 3.2. Nanogels and Their Descendants

Nanogels can be defined as non-ionic and ionic nanosystems that are made from physically or chemically cross-linked polymers, either hydrophilic, hydrophobic, or amphiphilic. The nanogel forming materials may also consist of polysaccharides and proteins, which are selected based on their biodegradable, less immunogenic response. These are made to be particularly successful in increasing the drug payload in the targeted region and controlling the propensity of all the other nanocarriers to leak the loaded bioactives. NGs possessing a diameter ranging between 1 and 1000 nm integrate the properties of hydrogels as well as nanomaterials [20]. A novel drug transporter ought to have two primary characteristics: it should carry the drug at the desired rate and it should successfully deliver the medication to the target site [21]. As a result, nanogels possess a number of sophisticated properties that can aid in the development of new delivery systems. A nanogel is defined as “a semisolid system consisting of a dispersion made up of either small inorganic particles or big organic particles contained and interpenetrated by liquid” by the United States Pharmacopoeia [22]. Nanogels have important qualities including better medicine penetration through the physiologic membrane and protracted drug release. For topical applications, nanogels are more stable as controlled drug carriers than traditional transdermal administration agents (oils, creams, and lotions) [23]. The main aim in phytopharmaceuticals is to deliver the active herbal drug without repeated administrations and with proper controlled release [24].

Nanogels are employed for both local and systemic drug action because of their natural ability to swell due to chemical alteration, allowing the drug to be released in the desired dosage form [13]. Nanogels can be used to make dermal patches, biosensors, and ionic drug delivery. Nanotechnology aims to increase the bioavailability of poor water-soluble herbal drugs via modulating their release [25]. These nanostructures can be distinguished on the basis of the composition of its lipids and polymer [26]. Drug delivery from carbohydrate-based nanogels can also be enhanced using lectin functionalization [27].

Chitosan, alginate, polyvinyl alcohol, and carbomers are some of the most commonly employed polymers for nanogel preparation [28]. Carbomer/Carbopol is a synthetic polymer that gels when its acidic pH is neutralized. The carbomer is initially heavily coiled, but after dissolving in liquid solvent, it partially uncoils. When the pH is adjusted to 7.0 using sodium hydroxide/KOH, the salt production causes complete uncoiling, thickening to create a nanogel. To transfer macromolecules such as genes, peptides, proteins, antigens, and oligonucleotides, nanoparticulates composed of chitosan hydrogel are widely used.

The drugs can also be released from these nanogels when they are stimulated. Only when calcium ions are added having affinity towards carboxylic group or the pH of the medium is reduced from 7.4 to 5.5 do PAA and PEG crosslinked hydrogels release oppositely charged proteins [29]. A triple-layered nanogel (TLN) encapsulating vancomycin was recently reported to degrade and release the antibiotic in the presence of lipase enzyme. A PEG shell, an ester linked with the phosphate group of the drug, a lipase responsive polycaprolactone crosslinked with the particles, and the layers make up the nanogel. The antibiotic is preserved in the core in a typical environment because the PCL (polycaprolactone) is intact. The PCL layer dissolves in the lipase of cellular microbial, releasing the antibiotic into the cell.

### 3.3. Nanogel Synthesis

The methods for creating nanogels can be categorized depending on size, polymerization method, and nanometer scale. The procedures for preparing gels and controlling their nanostructures are detailed in this section [30]. 

#### 3.3.1. Physical Techniques

**Figure 1:**
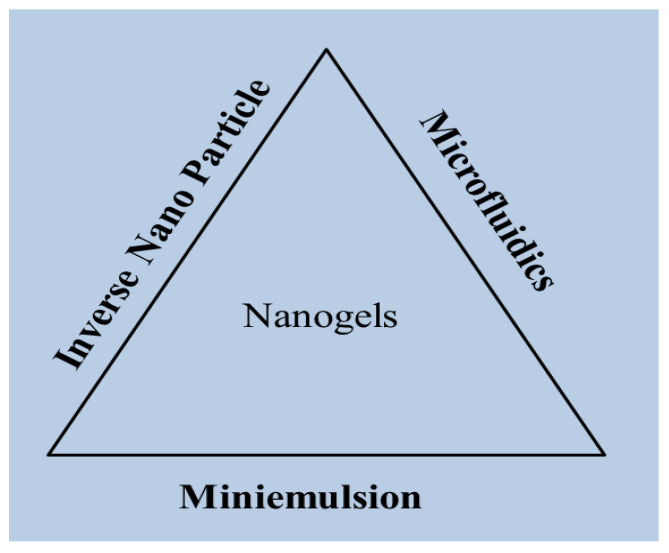
Different methods of nanogel preparation.

The miniemulsion technique, microfluidics, and inverse nanoprecipitation are some common physical techniques used to make nanogels (Figure 1). A water in oil emulsion is created during the miniemulsion process, which contains minute particles of oil-soluble surfactants present in a continuous organic phase. For droplet production in the microfluidic approach, glass chambers or a capillary tube constructed of polymer-like silica are used. The final system, called inverse nanoprecipitation, is the most systematic way to make aqueous nanogels and includes simply adding an aqueous polymeric solution to a miscible non-solvent [8]. 

##### 3.3.2. Crosslinking Method

**Figure 2:**
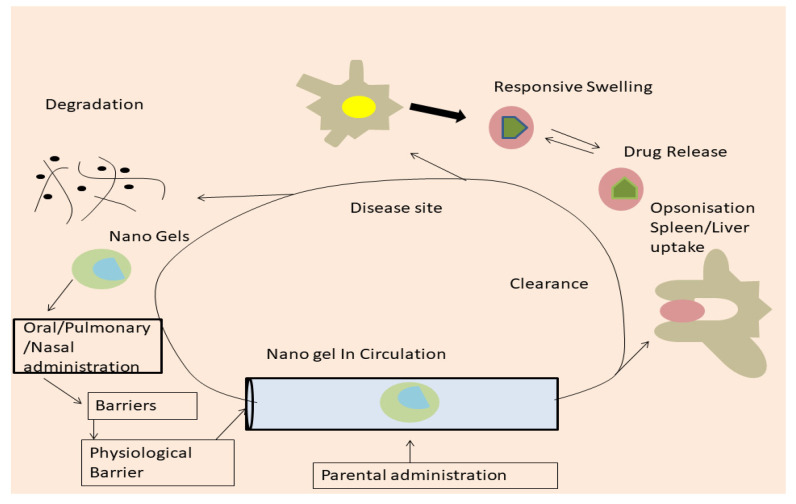
Mechanism of action of covalent crosslinking gel.

Covalent crosslinking is one of the best coupling methods for forming a gel network utilizing monomers with a reactive functional group with reduced molecular weight. Under in vitro conditions, the nanogels created by covalent bonding via crosslinking of their functional groups were highly stable, advantageous in the drug release and trapping process. In this crosslinking, chemical reactions such as free radical polymerization, Schiff base reaction, and other photoreactions may be employed [31]. In heterogeneous colloidal settings, such as w/o microemulsions, nanogels are prepared via crosslinking of preformed polymers [32]. This approach easily entraps small-molecule medicines and biomacromolecules into nanogels. The pH value, ionic strength, temperature, and ionization constant are all variables that affect the amount of the polymer to reach the required size. One of the major benefits of this method is that it produces disc or ellipsoid-shaped nanoparticles rather than spherical ones, avoiding phagocytosis. The microfluidic emulsification approach is paired with the crosslinking process, which results in a homogeneous dispersion of nanoparticles, thanks to new studies and manufactured products. Particle size is an essential consideration in drug administration because bioavailability is directly proportional to particle size as explained in Figure 2.

##### 3.3.3. Noncovalent Binding

Non-covalent binding is used to produce physical crosslinking nanogels, such as weak van der Waal forces, hydrophilic and hydrophobic contacts, and so on. These forms are less stable, and the gel’s sensitivity is strongly related to the composition temperature, crosslinking agent, and other factors. Nanogels, which lead to micelle production, have been shown to increase the solubility of highly lipophilic medicines by up to 30,000 times [33]. Polypropylene oxide and polyhydroxybutyrate, for example, are commonly utilized in the synthesis of biodegradable polymer micelles [34].

##### 3.3.4. Bioconjugation Technique

The free radical polymerization technique is a well-known and well-controlled method for producing nanogels of various sizes and shapes, including core-shell nanogels. In this bioconjugation technique, sub initiators such as functional initiatives, and micro initiators are used to prohibit functionalities from being included in the interior of nanogels, allowing for multivalent bioconjugation. Physically crosslinked systems that form under moderate conditions are more likely to be brittle than their covalently crosslinked counterparts due to fragile connections between polymer chains, such as hydrogen bonding, hydrophobic contacts, or non-covalent interactions [35]

## 4. Characterization of Nanogels

Nanogels should be adequately characterized before usage, and the methods listed below are typically deemed suitable for this purpose.

### 4.1. Dynamic Light Scattering

Dynamic light scattering (DLS) is used to determine the size distribution characteristics of nanoparticles in liquids. During various studies, light scattering is captured on a microsecond time scale. An effective hydrodynamic particle radius can be used to quantify the influence of the cross-linker, and the charges of the polymer chains on the size of the nanogel that is formed. DLS may also be used to determine how much nanogels swell in various mediums. It is worth noting that the DLS data may not account for the population of smaller polymer particles [36]. To fully comprehend the features of an object, a combination of analytical methodologies is frequently required. DLS was also used for analyzing the average diameter of particles and the polydispersity index.

### 4.2. Scanning Electron Microscopy

Electron microscopy provides a means for determining the morphology of the particle surface and the size. It can also be used to measure particles ranging from 50 to 80 nm and assess the morphological characteristics of nanogels.

### 4.3. Size-Exclusion Chromatography

SEC has been established as the gold standard for determining the molar mass averages as well as distributions of natural and synthesized macromolecules over the last half-century [37] When used in conjunction with a variety of detection methods, SEC can help us to better understand the physicochemical properties of polymers [38].

## 5. Properties of Nanogels

### 5.1. Degradability and Biocompatibility

Natural or synthetic polymers make up the nanogel. They do not collect in the organs forever because they are biocompatible and biodegradable. Nanogels can be made from chitosan, ethylcellulose, methylcellulose, and different polysaccharide-based polymers such as dextran, pullulan, and dextrin. Polysaccharides are carbohydrate-based polymers containing glycosidic connections that form a repeating pattern of monosaccharide units. These polymers are stable, non-toxic, hydrophilic, and biodegradable in their native state [39].

### 5.2. Swelling in an Aqueous Medium

Nanogels can swell in the presence of an aqueous solution since they are small, soft materials. It is regarded as the most important property that influences their drug delivery mechanism. It is based on the following:

#### 5.2.1. Structure of Nanogels

The chemical composition of the polymer chain, the degree of crosslinking, and, in the case of polyelectrolyte gels, the charge density are all things to consider. Environmental variables that are linked to aqueous medium properties, for example, pH, ionic strength, and the chemical composition of the ions are all critical factors in polyelectrolyte gels. Temperature is also a factor in the swelling of thermoresponsive gels [12].

#### 5.2.2. Higher Capacity for Drug Loading

Because of the swelling feature of nanogels, which allows them to absorb a considerable amount of water, they are projected to have a higher loading capacity than normal dose formulations. As a result, after inclusion and loading, the water will have enough cargo capacity to hold salts and biomaterial. Loading can be done in various ways such as, but not limited to:

1. The interaction of hydrophilic chains with the hydrophobic sections of a polymer, as well as the dissolution of hydrophobic molecules in a hydrophilic vehicle, is referred to as physical entrapment. The creation of a dense drug-loaded core is caused by the covalent bonding of bioactive molecules.
2. Self-assembly under controlled conditions, similar to that of polyelectrolyte-based nanogels. A high loading efficiency is achieved by the interaction of oppositely charged electrolytes. Composition, molecular weight, potential interactions between the medication and the polymer used, and the varied functional groups in each polymeric unit are all factors that contribute to the higher loading capacity.

#### 5.2.3. Particle Size and Permeability

The permeability of nano delivery systems can be dramatically improved by slight changes in particle size, surface charge, or hydrophobicity. Despite the fact that nanoparticles can pass through tissues or damaged endothelium, as well as through a specific transport mechanism in some situations, crossing the blood–brain barrier (BBB) has proven difficult. Nanogels with diameters ranging from 20 to 200 nm may be small enough to pass through BBB without creating too much disruption.

#### 5.2.4. Colloidal Stability

Because they have lower CMC, lower dissociation rates, and longer drug retention, nanogels, also known as polymeric micellar systems, are more stable than surfactant micelles. Various colloidal drug carrier technologies for regulated intravenous drug delivery, such as liposomes, fat emulsions, and nanoparticles, are widely recognized. Under in vivo conditions, the nanogels can be functionalized and modified to lower protein binding and to avoid detection by the phagocytes, and hence avoid removal of the nanosystems from the systemic circulation through opsonization and phagocytosis [40]. 

### 5.3. Stimuli Activated Nanogels

#### 5.3.1. pH-Stimulated or Responsive Nanogels

**Figure 3:**
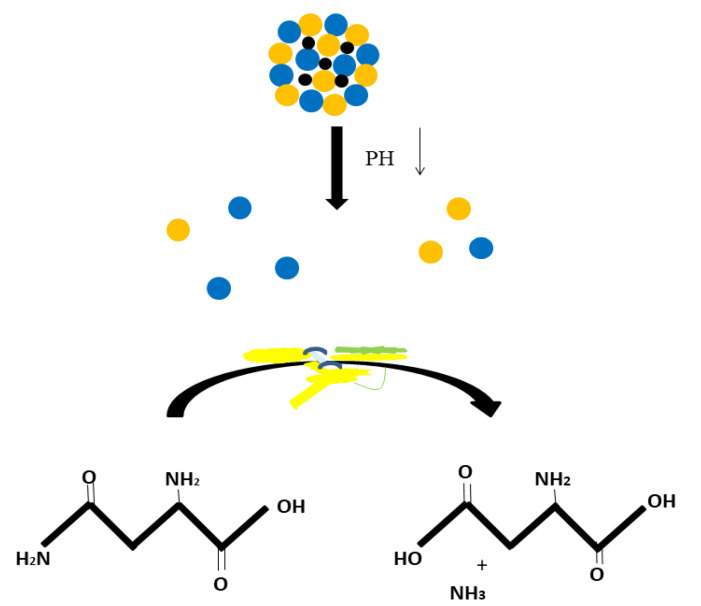
pH-responsive nanogels may be activated by an increase in pH caused by environmental change pH responsive nanogels.

pH-stimulated or responsive nanogels with a slightly acidic or basic polyelectrolyte chain may have an electron-withdrawing or electron-donating group. These pH-responsive nanogels may be activated by an increase in pH caused by environmental changes because of their composition as shown in Figure 3. Positively charged nanoparticles strongly attract blood serum, which causes aggregation and rapid particle clearance, resulting in a reduced effect. Meanwhile, specific negatively charged nanoparticles have a longer plasma half-life than positively charged nanoparticles and are resistant to protein binding. The mechanism of action is based on the activity of proton uptake by the gel and its ionization group, which primarily consists of two steps: rapid binding of cations or anions to the gel’s surface and diffusion of these ions into the gel’s network. Because it is utilized to define the pH microenvironment or the body location where the trapped medicines would be released, pHc is an important parameter for pH-responsive nanogels for drug delivery. The pHc of nanogels is frequently calculated using the pKa of weakly acidic groups or the pKb of weakly primary groups across the polyelectrolyte chain. Anionic pH-responsive nanogels expand at a pH higher than the pKa of weakly acidic groups, while cationic pH-responsive nanogels expand at a pH lower than the pKb of weakly basic groups [17]. Dextran nanoparticles are also known to play an important function in the delivery of insulin peptide via several catabolic routes (such as glycolytic pathways) at a specific pH [41]. The polymer used in the system is insoluble at neutral pH. As the pH decreases, the polymer expands, and the drug begins to depart the system. Glucose is converted to gluconic acid by the enzyme glucose oxidase, which lowers the physiologic pH. Because of the swelling action of pH-sensitive polyacrylic acid chains, temozolodine had controlled release kinetics [42].

#### 5.3.2. Temperature-Stimulated Response

Temperature-sensitive nanogels tend to swell and de-swell in response to temperature variations due to their sensitivity to temperature. Their classification is divided into two categories based on the low critical solution temperature: positive and negative responsive systems. Nanogels that respond negatively to temperature are predominantly constituted of a polymer (poly(N-isopropylacrylamide) (pNIPAAm)) and have a vital temperature in aqueous solution; the temperature is inversely proportional to their particle size. The release of confined medication in reaction to temperature change is at the heart of all nanogels that are temperature-responsive and are utilized or created for the efficient delivery of drugs. Almost all nanogels that are temperature-responsive for drug administration are built for releasing limited medications in reaction to temperature increases due to higher atmospheric temperature of specific diseased sites at cellular level. Furthermore, following the administration of hyperthermic stimulation to the sickness site, the drug is swiftly discharged in the specified space. A brief cold-shock therapy can also responsively cause release by generating a temperature reduction.

**Figure 4:**
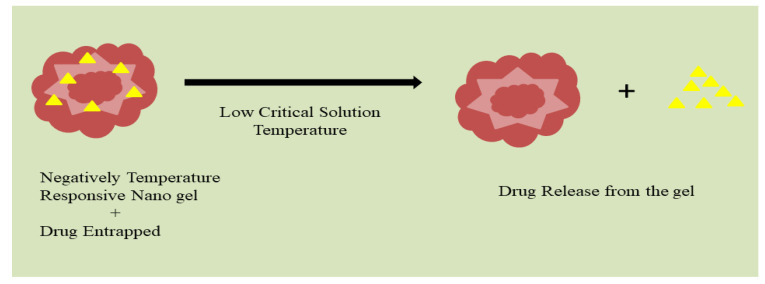
Temperature responsive nanogels and drug release from the thermogel.

Temperature stimulus nanogels, for example, were employed as anticancer drug delivery systems by physically destroying 200 nm endosomal vesicles within cells via a sudden volume expansion triggered by an externally regulated drop in temperature. Polymers that respond to temperature, such as polyisopropylacrylamide and polyvinylcaprolactam [41], have a lower critical solution temperature (LCST) in the aqueous phase of roughly 32 °C [43]. Nanogels generated from these temperature-sensitive polymers expand at low temperatures and collapse at high temperatures, showing a transition temperature near physical response at volume phase (Figure 4). Because of their unusual behavior, these nanogels are attractive for biotechnological applications [44].

#### 5.3.3. Enzyme-Responsive Nanogels

Enzymes regulate most activities in the human body, and enzymes can respond to or degrade a wide range of molecules in living organisms. Enzyme-responsive nanogels, in general, have moieties that have the ability to change the functionality after enzyme activation. Chemical and/or physical qualities can alter as a result of these modifications. Natural materials, such as peptide sequences or polymers, are frequently used to create enzyme-response functions. The biocompatibility, selectivity, and cognizability of enzymes make enzyme-responsive nanomaterials very appealing. Enzyme-responsive nanogels possess a lot of potential in biomedical applications because of these properties [20].

#### 5.3.4. Multiresponsive Nanogels

External inputs trigger all-purpose nanogels, which can then respond to a variety of stimuli. The two types of multi-responsive nanogels are NGs that react to each external stimulus separately, also known as the “A or B” type, and nanogels that respond to just outer stimuli when they are present, also known as the “A and B” type. Multi-responsive nanogels are typically generated by integrating numerous stimulus-sensitive polymer components into their network via a random co-polymerization method [45].

## 6. Nanogels in the Biomedical Field

Nanogels are promising biological materials made up of dispersions of hydrogel nanoparticles based on crosslinked polymeric networks, which are minimal owing to their high drug encapsulating capacity, homogeneity, adjustability, and ease of production [46]. Chemotherapy, diagnostics, organ selection, and bioactive drug delivery are just a few of the applications for nanogels. Nanotechnology products have become increasingly relevant in biomedicine, resulting in nanobiotechnology as a hybrid science [47]. Nanomaterials are used extensively in nanobiotechnology, including diagnosis, drug delivery systems, prosthetics, and implants. Nanoscale materials work well with biomedical equipment since most biological processes are nanoscale. Materials used to make nanotechnology products include inorganic and metal nanoparticles, carbon nanotubes, liposomes, and metallic surfaces [48,49]. Nanotechnology has been at the forefront of recent technological advances in disease diagnostics, medicine development, and drug delivery. Nanomedicine is a word used to describe the use of nanotechnology in the treatment, diagnosis, monitoring, and control of biological systems [50].

### 6.1. Drug Release from Nanogels

#### Nanogel Drug Encapsulation Techniques

The majority of the time, nanogels are employed to deliver pharmaceutical substances. A successful nano delivery system will have a significant drug-loading capacity, which will reduce the number of transporters needed. Drugs can be introduced into nanogels using different methods [51].

Covalent Conjugation

Biological agents can be covalently coupled with nanogels that already exist or during nanogel manufacture. Acrylic groups are changed with enzymes and copolymerized with acrylamide in an inverted nanoemulsion or an aqueous containing solution to form a nano-sized hydrogel, for example [52]

Physical Entrapment

Because of the physical trapping of siRNA in HA nanogels, proteins might be incorporated into cholesterol-modified pullulan nanogels. Many nanogels have a hydrophobic chain formed when hydrophobic molecules are linked to nonpolar domains. Prostaglandin E2 was, for example, solubilized in cholesterol-modified pullulan nanogels. Another work used noncovalently integrated N-hexylcarbamoyl-5-fluorouracil in crosslinked nanogels of N isopropylacrylamide (NIPAAm) and N-vinylpyrrolidone (VP) copolymers (PNIPAAm/VP) (HCFU). Physical entrapment is affected by the sizes of nanocarrier holes and enzyme molecules. Others have concentrated on the functional groups found on enzymatic sites [53].

Self-Assembly

The thermodynamic stability of biomolecular ensembles with regular topologies and cognitive functions has been proven in biological self-assembly. Self-assembly is described as the spontaneous, autonomous, and reversible grouping of molecular units into structurally stable and well-defined aggregates in which errors are energetically rejected. Self-assembly is an autonomous organization of components that are aggregated into a structurally well-defined structure [54]. It has a host of advantages, including:

- economically friendly,
- adaptable and simple,
- process occurs at the state’s adiabatic minimum, leading to structures that are robust and stable.

Other attractive and repulsive interactions including electrostatic, van der Waals, Coulomb contacts, hydrophobic forces, and hydrogen bonds, balance the system’s thermodynamic minima, resulting in self-assembly. Many molecules are held together by non-covalent interactions, hydrophobic associations, and electrostatic interactions after they self-assemble via diffusion. These interactions are weak on their own, but because of the enormous number of contacts involved, they dominate the structural and conformational behavior of the assembly.

While electrostatic attractions quickly join oppositely charged polysaccharides, interactions with neutral polysaccharides are weak or non-existent; yet, chemical modification may necessitate assembly. Because the polysaccharides are very water-soluble, hydrophobic interactions result in the creation of nanoparticles. This type of amphiphilic polymer can be used in three ways.

- A hydrophobic backbone with hydrophilic chains grafted on it (grafted polymer).
- A hydrophilic backbone grafted with hydrophobic chains.
- Alternately, with hydrophilic and hydrophobic segments (block polymers) [55].

### 6.2. Mechanism of Drug Release from Nanogels

#### 6.2.1. Diffusion Technique

Doxorubicin is released by diffusion from pluronic block copolymer-based stable hydrogel nanoparticles. This basic technique and principle are applied in a range of nanomedicines, including polymeric micelles previously tested in humans. Some examples of coating materials include small molecules such as folic acid or galatose, petides such as RGD or ATWLPPR, other typologies of proteins or antibodies, and so on.

The breakdown of these nanogels and the elimination of empty carriers prompted the release of encapsulated substances such rhodamine 6G, a fluorescent dye, and Doxorubicin, an active metabolite. The release of Doxorubicin was considerably boosted due to the grafting of the dimethyl aminopropyl group on glycol chitosan nanoparticles, which makes them sensitive to pH stimuli. Because of pH sensitivity, the mesh size of a diethylamino ethyl methacrylate cationic nanogel for medium-sized molecule release has changed drastically.

#### 6.2.2. Movement of Ions 

Nanogels are gaining popularity as a way to release natural compounds in reaction to changes in cues at a specific action site. In the presence of a glutathione tripeptide, which would be commonly found in cells, disulfide covalently bonded POEOMA nanogels decomposed into a water-soluble polymer [56]. The cellular accumulation of an NTPs prescription given with nanogels could be explained by cell membrane-triggered release of negatively charged medicines into complexes with cationic nanogels.

#### 6.2.3. pH Stimulative Techniques

The on and off analytic activity is scavenged by platinum nanoparticles with nanogel and crosslinked poly(2-N,N-diethylaminomethacrylate) core and PEG in the acidic skin pH [57]. When the external pH is low, the polymers methacrylic acid and ethyl acrylate create insoluble 3D forms. As a result of polymeric chain repulsions, increased pH ranges cause acidic groups to ionize, resulting in a unique procaine hydrochloride release profile. The rate of solubility of the temozolomide drug is controlled by the swelling effect of pH-sensitive polyacrylic acid chains.

#### 6.2.4. Photochemical Internalization and Photoisomerization

The discharge of medicinal chemicals or APIs into the cytoplasm is influenced by the oxidation of cellular obstacles, such as endosome barrier walls, produced by photosensitized packed nanogels, which produce solitary oxygen and reactive oxygen molecules. Biological carriers can be liberated from polyelectrolyte hydrogels transporting biological agents due to electrostatic interactions in reaction to environmental changes. In an azo-dextran nanogel packed with aspirin as a model pharmaceutical, cis-trans isomerism of azobenzene by photoregulation at 365 nm demonstrated that the e-configuration of the azo group generates a better medicinal liberation profile than the z-configuration [58].

## 7. Nanogels as a Therapeutic Carrier Agent

Nanogels are utilized to deliver particular proteins to specified locations. Nanoparticles are revolutionizing the field of medicine delivery. These drug nanocarriers have the potential to improve the medical efficacy of a treatment by adjusting the release and stability of a medication, extending the circulation time of a drug, and protecting it from phagocytic cell clearance or premature degradation. Nanoscale carriers can also be engineered to concentrate in tumor cells and tissues by improving absorptivity and creating a retention effect or actively targeting tumor-associated antigens with ligands. In vivo studies have shown that nanogel-based drug delivery formulations improve the therapeutic impact of antifungal, anticancer, and anti-diabetic drugs while also improving the ease of administration [59].

### 7.1. Chemotherapy (Anticancer Therapy)

Cancer has been treated with various molecular nanogels. Chemotherapy drugs that are incorporated into nanogels boost not just bioavailability but also absorbency and retention. Nanogels are being used in cancer treatment to improve the delivery of drugs. When Ayurvedic nanomedications are employed in cancer therapy, they give targeted medication that is therapeutically effective and has few side effects. This notion has gained widespread acceptance, although it has substantial downsides due to a lack of technology available, which could jeopardize the validation process. Cancer nanotechnology developed by Ayurveda (size range 1–100 nm) can transform cancer treatment. For example, *Satureja khuzistanica* jamzad, an essential oil, has shown significant activity as an anticancer agent as well as an antimicrobial agent. Doxorubicin-loaded nanogels is another class of drug delivery system which is also found to have a vital role in performing anti-cancer activity [60]. Some herbal nanostructures used in the treatment of cancer are liposomes, reconstituted high-density lipoprotein (rHDL), micelles, dendrimers, nanogels, nanoemulsions, and exosomes.

### 7.2. Autoimmune Disease

The ability of the medicine delivery strategy to specifically suppress leukocytes that trigger the autoimmunity response is critical for immune disorder treatment [61]. Nanogel delivery methods have been intensively researched for the goal of integrating immunosuppressive medications, as nanogels can enhance immunosuppression by targeting antigen-presenting cells that leads to abnormal functioning of normal body cells. By loading liposomes with a diacrylate ending polymer of polyethylene glycol, the gel containing mycophenolic acid complexed cyclodextrin (not methylated) was created and evaluated [62]. This form of medicine delivery system will improve patient compliance while also delaying the start of renal impairment, a typical lupus consequence [63].

### 7.3. For Local Anesthesia

Injectable hydrogel systems are attractive prospects for prolonged, targeted anesthetic delivery due to their ability to localize drug delivery through less invasive injections of hydrogels. In vitro and in vivo studies of many responsive hydrogel systems for the delivery of bupivacaine, ropivacaine, and lidocaine revealed regulated release, extended analgesic effects, and low cytotoxicity. Local anesthetics are a type of anesthetic used to generate a physiological condition that decreases or eliminates pain. At high pH, a derivative of –NH_2_ salt nanogel demonstrated a considerable release rate for procaine via lipophilic and gaseous bonding delivery. The deprotonating of the acid on the nanogel generates an increase in pressure and, as a result, swelling of the entire system, allowing the procaine to be ejected [64].

### 7.4. Stopping Bleeding

A protein molecule in the solution that has been used to make a nanogel has been used to control bleeding even in major wounds. On the nanoscale, the proteins have a method for self-assembling into a biodegradable gel. For example, micronized sacchachitin is effective in the wound healing process [65].

### 7.5. Anti-Inflammatory Action

Anti-inflammatory medications can be delivered to individuals with skin problems using nanogels. For effective drug administration in dermatitis, a skin penetrating nanogel system composed of a surface of double layered nanostructured particles and an emulsifying agent has been created. In this SPN, polylactide glycolic acid or chitin were employed to construct bilayered nanoparticles (NPS), while oleic acid (NPSO) was used to create a modified surface. To create nanogels, hydroxypropyl methylcellulose (HPMC) and carbopol were used after reaching the required viscosity. These investigations have shown that drugs can be given efficiently through the skin in the event of skin irritation. Diclofenac is a type of non-steroidal anti-inflammatory drug (NSAID) that is commonly used to treat arthritis. Hydrogels of diclofenac sodium self-assembling gel provided improved anti-inflammatory results. In addition, nanogels containing cinnamon oil and cinnamaldehyde are found to have good antimicrobial activity [66].

### 7.6. Ophthalmological Difficulties

PVP/PVAc is a polyvinyl pyrrolidone/polyvinyl acetate combination wherein gamma radiation-induced polymerization is used to make a pH-friendly nanogel. It has been used to incorporate alkaloids to keep them at a desired concentration at the action site for longer [67].

### 7.7. Neurodegenerative Diseases

As the population ages, neurodegenerative disorders including Alzheimer’s disease, Parkinson’s disease, multiple lateral sclerosis, and stroke are on the rise [68]. Nanomedicine is a fast-growing discipline that offers leading advancements in the detection and treatment of life-threatening human diseases [69]. The delivery of poly-nucleotides to the brain has been demonstrated using nanogels [70]. For the prevention of neurological diseases, parenteral administration of oligonucleotides (ODN) to the CNS is advocated [71]. Injected macromolecules are quickly eliminated from circulation after failing to cross the BBB. Nanogels encapsulated in or coupled to spontaneously negatively charged ODN generate a stable aqueous polyelectrolyte compound with particle diameter below 100 nm that readily penetrates across the blood–brain barrier. The transport efficacy of the nanogel is significantly enhanced when the surface is changed with transferrin or insulin [50].

### 7.8. Diabetes

Insulin-loaded nanogels appear to be more effective than free insulin at maintaining blood glucose levels and reducing blood sugar swings. Insulin-loaded nanogels lowered blood glucose levels in diabetic rats by 51% from baseline in vivo studies. It consisted of nanoparticles with opposite charges that are attracted to one another. The gel did not essentially dissolve, and the nanoparticles may not spread throughout the body [71].

### 7.9. Intranasal Administration

Nanogel drug delivery technologies hold a lot of potential for resolving some of the obstacles associated with drug delivery. Nanogels could transport and distribute drugs through the nasal mucosa because the mucosa quickly absorbs them [71]. Nasal vaccination with nanogels is an innovative technique to limit disease progression.

### 7.10. Vaginal Drug Delivery

Since ancient times, the vaginal route has been used to administer drugs. Antibacterial nanogels have been used in vaginal nanogels to prevent various vaginal infections. They are also suitable for treating vaginal soreness, discharge, and a variety of other sexual disorders. Vaginal nanogel has several disadvantages, such as that it should not be used during menstruation or pregnancy. Antiviral vaginal nanogels have been shown to reduce the risk of HIV infection in women. The use of tenofovir vaginal gel in HIV prophylaxis has been investigated. Tenofovir gelatin nanoparticles were made using a two-step desolvation technique. HPMC K15M was used as both a connecting co-polymer and a viscosity ingredient in this study. Ovalbumin and lysozyme, two proteins found in hen egg white, have been synthesized as nanogels wherein a solution of ovalbumin and lysozyme with pH 5.3 adjusted up to pH 10.3 was mixed and heated. The core was built of lysozyme, while the shell was made of ovalbumin because the nanogels are spherical and have a core-shell shape. Intermolecular hydrophobic interactions, hydrogen bonds, and disulfide connections bound these proteins in denatured states. The charges on nanogels can be changed by adjusting the pH of the solution while the surface structure of the nanogel can be stabilized using electrostatic repelling force. Apart from that, the solutions of these nanogels are quite stable for long periods of storage, and they can also be stored as a lyophilized powder, which makes them more useful. Nanogels containing non-microbial medicines were utilized for inhibiting different infections in the vagina [72].

### 7.11. Gene Therapy and Tissue Engineering

Tissue engineering and gene therapy both rely heavily on nanogel-based formulations. They are also employed to deliver enzymes, DNA, and proteins to specific regions so that the desired effects can be achieved. Modifying polymers to transport enzymes and proteins using artificial chaperons is commonplace. Pullulan is also chemically changed by conjugating cholesterol moieties, and the functionalized molecules then self-assemble in water to create NGS as small as 30 nm. These nanogels are used for bone regeneration because of their great biocompatibility [73].

### 7.12. Antifungal Agent Transporter

Physicians and patients prefer mostly the stratum route for fungal infections. Using reconstructed chemistry and hence the wet edge process, a fluconazole-chitin nanogel was created. Fluconazole-chitin has a regulated release pattern that allows fluconazole to be available for a long time, allowing for successful fungus therapy [74]. 

## 8. Herbal Products

Natural products benefit human health because they contain phytochemical components that have active therapeutic capabilities. Plants such as fruits, vegetables, cereals, legumes, and other valuable plants produce these chemicals. Some plants can inhibit the growth of cancer-causing agents. Phytochemical compounds are isolated from plants and employed in medications with the help of nanotechnology [75]. Herbal medicines have been used to treat various disorders since the Middle Ages and are an essential aspect of integrative and indigenous medicine. Because several medications already in use are derived directly or indirectly from plant sources, scientists have started adopting herbal drugs in recent decades. Herbal medicines are becoming immensely popular because they have limited adverse effects on human health, and are relatively safer and less expensive than conventional medications. Natural products are excellent sources of bioactive chemicals and are widely regarded as contemporary medicine’s most successful discovery [76].

Because of their excellent response in vivo, high drug loading capacity, excellent absorption, and cellular like features, nanogels have shown significant promise for the transport of a wide range of medications to various organs of the body [63]. Aloe vera is a natural component that has been used for millennia to heal skin problems and has a high level of acceptability among the general public. The use of co-emulsifiers can improve drug penetration and permeability, and studies have shown that employing nanogels in combination with co-emulsifiers improves cutaneous delivery qualities, and this article explains how to build a formulation using pharmaceutically acceptable constituents.

Herbal medicine is frequently defined as “therapeutic techniques that have existed for many years before the emergence and distribution of modern pharmaceuticals” [8]. Herbal medicine is a discipline of medical sciences that employs medicinal plants in treatment and is the subject of extensive research. In today’s world, herbal drugs derived from ancient herbaceous drugs are rationally recognized as other drugs for treating and curing most communicable and non-communicable disorders such as cancer and diabetes. Herbal remedies have played a significant part in establishing the foundation for today’s modern pharmacopeia. Herbal medicines are preferred over modern pharmaceuticals since they have fewer side effects and are healthier patient alternatives. Around 85% of the world’s population used herbal treatments to cure skin diseases, viral and fungal infections, diabetic and hypersensitive reactions, and so on. Despite their efficacy in vivo, they are under-utilized in clinical practice for a variety of reasons, including solubility issues, bioavailability issues, and high dose needs. If used correctly, they can be used in routine medical activities. As a result, the dose of herbs used for pharmacological activity is reduced; nonetheless, the accessibility and cost-effectiveness of these traditional medicines make them more acceptable as a substitute for modern medications. Highly monodisperse AgNPs silver nanoparticles made from the leaf extract of Tulsi are well-reported therapeutic agents [77]. Some examples of herbal products for the treatment of neurodegenerative diseases are curcumin, quercetin, resveratrol, piperine, gallic acid and epigallocatechin-3-gallate, and ferulic acid, among others [61].

## 9. Herbal Drug Formulations

### 9.1. Nano-Capsules

Herbal nanocapsules are natural drug treatments that comprise nanoshells synthesized from a pure polymer. These nanocapsules are used to supply drugs to a particular region in a regulated and targeted manner. Poly-e-caprolactone (PCL), poly(lactide) (PLA), and poly(lactide-co-glycolide) [78] are the polymers utilized to make natural nanocapsules (PLGA). Nanocapsules are being used in natural medicinal drugs due to their small size and excessive surface area to aspect ratio, in addition to the fact that nanoparticle drug therapies enhance pharmacokinetic and biodistribution of therapeutic agents [37].

### 9.2. Herbal Nanoparticles for Cancer Therapy

Ayurvedic nanomedicines in cancer therapy allow for tailored medication administration with improved therapeutic efficiency and fewer adverse effects and has the potential to alter the way cancer is treated, diagnosed, and detected. Repeated chemotherapeutic therapy has led to tumors resistant to these agents, which is now one of the leading causes of cancer [79]. As a result, identifying natural compounds that target and multiply signaling pathways, as well as growth inhibition, is highly beneficial [80]. Without a doubt, cancer treatment necessitates the administration of drugs with low toxicity to surrounding tissues and high therapeutic efficacy. Nanogel technology ensures all of these advantages.

### 9.3. Nano-Tablets

Populations can utilize the purifying power of herbal water pills containing nanoparticles in developing countries for safe drinking water. This tablet has been created with Brahmi (*Bacopa monniera*) extract on a tiny ceramic disc loaded with silver or copper nanoparticles, which is placed inside a water vessel and can cleanse water for up to six months. For controlled and targeted delivery, nanotablets containing herbal medications are used. The anticancer effect of ayurvedic bhasmas coated nanotablets is being investigated [81].

The term ‘bhasma’ contains nanoparticles in its formulations. Many specialists are astounded to learn that India has a 5000-year-old medical system [82]. For ages, the bhasmas have been used in Ayurveda for the treatment of numerous diseases in the form of nanotechnology [83]. ‘Rasayana’ (immune modulation and anti-aging characteristic) and ‘yogavahi’ (drug carrying ability and targeted drug delivery) are two prevalent properties in ayurvedic bhasmas [84].

Herbal nanogels are hydrogel nanoparticles with diameters ranging from 10 to 100 nm that are more effective in controlling and targeting the drug release. Ayurvedic nanogels are the most effective treatment for our health, particularly metabolic health [85]. It is the quickest and safest approach to lose weight without causing any adverse effects, and it lowers fat in the abdomen, arms, legs, thighs, and double chin. It may also act as a heart tonic that can aid with a variety of cardiac problems. Biopolymers with herbal medications such as curcumin and natural extracts of caffeine, laminaria, and ivy make up the nanogels. This nanogel penetrates deeply into the skin, acting directly on accumulated fat to reduce it [86].

### 9.4. Nanoemulsions

Nanoemulsion qualities are influenced not only by the composition but also by the manner of preparation. These nanoemulsions can be used to transport medications to cells and cancer therapy and disinfection. Nanoemulsions were first developed about 20 years ago, mainly for the synthesis of nanoparticles. Today, nanoemulsions are primarily used in pharmaceuticals and cosmetics. Ayurvedic nanoemulsions with a diameter of 20–200 nm have a wide range of applications. They can be used in transdermal delivery systems and are non-toxic and non-irritating. As a result, they improve the drug’s solubility and bioavailability [87].

### 9.5. Nanopaste and Nano Pure (Nano-Air Purification)

Aloe vera-based herbal nanopaste is now being researched for the treatment of osteoporosis. As the nanoparticles are distributed sequentially and continuously stimulate the surrounding bone cells, this nanopaste strengthens the bones after surgery and has a long-lasting effect. Nanotechnology can also improve long-term air quality, availability, and viability of air resources, for example, through better filtration that allows for more air re-use, recycling, and purifying using herbal formulations [88].

### 9.6. Percutaneous Uses of Herbal Medicaments as Nanogels

When compared to traditional techniques, dermal dosing systems have demonstrated to be more effective, avoiding the drug’s first-pass metabolic effect and aligning the patient’s preference with that of drug release [89]. The limited absorption of the medicine through the skin is a problem that no other route of delivery can overcome. In this area, nanogels are being studied in order to obtain the best skin penetration while also incorporating additional functions, such as drug release reaction to environmental stimuli, pH, and other parameters [20]. For example, curcumin-loaded chitosan nanogels had great skin permeation and significantly improved curcumin absorption.

### 9.7. Herbal Nanogels for Oral Usage

Oral administration is the standard method of administration for many therapeutic treatments. On the other side, oral dosing has some disadvantages, including first-pass metabolism, gastrointestinal degradation, and limited bioavailability. Although oral delivery has a large market potential, it is confined to a small number of chronic conditions due to the negative side effects of oral drugs [90]. Due to its non-poisonous qualities, excellent drug release, or better systemic release, nanogels have made significant progress in buccal natural fauna formulation. Oral administration is the preferred method of administration for many therapeutic drugs. First-pass metabolism, gastrointestinal breakdown, and limited bioavailability are all disadvantages of the oral route of administration. Although oral administration has a large market potential, significant side effects of oral drugs, it is limited for so many medical illnesses. Because of their non-toxic action, high bioavailability, and faster release rate in the system, nanogels have achieved remarkable progress in oral herbal drugs.

Curcumin is the most frequently used and extensively researched natural substance [91]. In cancer research, curcumin is the most commonly employed herbal ingredients. Curcumin is better entrapped in inverse miniemulsion alginate aldehyde gelatin nanogels. The use of acetone containing curcumin to precipitate nanogels improves encapsulation in the crosslinked polymer network. End–end hydrogen bonding forms a better encapsulation when the hydroxyl group of curcumin interacts with not reacted hydroxyl functions in the alginate aldehyde [92]. Curcumin encapsulation in nanogel enhances its solubility, improving drug loading efficiency to efficacy index levels for buccal administration. It has been discovered that substantial medicament loading should not affect the herbal nanogel’s encapsulation stability. Another crucial feature of a successful delivery vehicle system is the nanogel’s ability to remain stable in vivo. When compared to non-crosslinked polymeric nanogels, studies show that crosslinked polymeric nanogels have more stable characteristics.

## 10. Other Nanogels

Nanogels are one of the most prominent nanotechnology strategies for successful drug administration both within and outside the body, as well as for topical treatment (Table 2). Nanogels have features that allow them to transport materials such as DNA, proteins, oligonucleotides, RNA, dyes, quantum dots, and chemical agents such as diclofenac to the target region [93]. Its nano-sized structure has shown to lower drug molecule toxicity and to offer regulated drug release at the target site, hence increasing drug bioavailability [94]. Herbal medicine’s efficacy is determined by the cooperative action of all of its vital ingredients. Because maximal herbaceous medications contain insoluble parts, they have low bioavailability and a high systemic clearance [95]. These medications’ nanogel compositions help to overcome these constraints. A variety of nanogels containing herbal medicines are listed below.

*Eupatorium adenophorum* (Asteraceae) leaves are utilized as an antibacterial, painkiller, and lesion therapy in Ayurveda [96]. Negi and co-workers incorporated a methanolic extract (1% *w*/*w*) of *E. adenophorum* leaves within a carbopol 934 gel. The greenish herbal gels formed provided efficient anti-inflammatory efficacy in a carrageenan-induced rat paw edema model [97].

Bronchitis, asthma, fever, skin ailments, and epilepsy have all been treated using leaf preparations of *Cleodendron infortunatum* in the past. The leaf extract was turned into a nanogel by Das et al. using the synthetic polymer carbopol 940. The 2.5% extract gel had a good anti-inflammatory effect and did not irritate the skin [98].

Bolleddu et al. reported that a gel made from methanolic *Albizia lebbeck* extracts possesses anti-inflammatory and analgesic effects [99]. The sodium alginate and carboxy-methyl cellulose nanogel had superior permeation than carbopol 934 and other combinations. Paul et al. studied the anti-inflammatory properties of a root extract nanogel [100]. Aqueous root extract was initially encased in silver nanoparticles before being transformed into a gel using a paraffin wax basis. Heat-induced denaturation of bovine serum albumin was effectively prevented by the gel. According to the researchers, a non-irritant nanogel containing *Sesbania grandiflora* leaf (ethyl acetate extract), Carbopol 934, and sodium CMC can be utilized to treat a variety of skin inflammations [22].

*Lantana camara* leaves offer anti-hemorrhoid and anti-inflammatory properties. Carbopol 934 was used to make gels from the two strengths of extracts (2.5% and 5%) [101]. Pawar and Shamkuwar discovered that the 2.5% extract gel was superior to the 5% extract gel in terms of physicochemical characteristics [102].

The ethanolic extracts of *Butea frondosa* stem bark have analgesic and anti-inflammatory effects. Shankar et al. produced a better gel formulation with carbopol 934 and DMSO, and after 8 h found diffusion and permeation percentages of 92.37 and 98.29, respectively [103].

To generate a semisolid dosage, researchers mixed *Boswellia serrata* (kunduru) extracts with *Withania somnifera* extracts. Because of its ability to inhibit 5-lipoxygenase, Boswellia serrata (pentacyclic triterpenes) is anti-inflammatory and anti-arthritic. *Withania somnifera* contains withaferin A, a cell-permeable steroidal lactone that acts as an anti-inflammatory and anti-arthritic [104].

A separate approach was used to investigate the bactericidal efficacy of glycolic extracts from pomegranate, apricot, and green tea. The astringent and antibacterial properties of pomegranate can be attributed to the fruit’s alkaloids and gallic tannins. Apricots have neuroprotective and remineralizing properties in addition to the same purpose. Green tea has antioxidative, anti-inflammatory, and chemoprotective properties, among other things. Green tea gallic acid extract gel was found to be effective against *Staphylococcus aureus, Pseudomonas aeruginosa*, and *E. coli*. This activity is considered to be caused by the catechins found in green tea [105].

Jadhav et al. investigated the antibacterial activity of ethanolic extracts of Tridax procumbens against Staphylococcus aureus [106]. Significant antibacterial activity was discovered in carbopol 940 gel containing 1% extract [107].

Numbness of the tongue and gums is caused by mastication of the leaves and petals of *Spilanthus acmella*, also known as Akkalkara. It is also utilized as a pain reliever or also used as anti-proliferation medication. Researchers investigated the effects of combining ethanosomes with the herbal extract in a muco-adhesive gel to treat pain, cavities in teeth, and buccal ulcers [108].

*Aloe barbadensis* is often applied to hasten the cascade process (various levels related to recovery: wound reduction by contracting, and back to normalized physiological functional barriers), to enhance immunity (by enhancing activation of B-cells and other defense mechanisms), and to treat various fungal infections. In rats with skin excision wounds, Khan et al. discovered that an aloe vera-carbopol 934 nanogel formulation promoted wound contraction. This ability is aided by the presence of mannose-6-phosphate in the leaf extracts. Mannose has been demonstrated to boost fibroblast activity and collagen production [109].

Misal et al. used *Cassia alata* Linn. to create a nanogel wherein the anticancer, anti-proliferative, reduction in microbial activity, dermal infections, and cascade process of the botanicals was enhanced [102]. *Cynodon dactylon* Pers. possesses antiviral, antidiabetic, antifungal, antibacterial, and antiulcer properties in addition to wound healing antioxidative activity. In rat paw edema caused by carrageenan, the polyherbal gel was found to have a higher anti-inflammatory effect than the individual gels [110].

## 11. Challenges and Opportunities for the Future

Nanogels are a valuable, novel, and successful medication delivery approach that addresses both traditional as well as modern healing concerns, including specific side effects and limited stability. Each new study claims that it has discovered unique polymeric mechanisms and mechanistic views with potential therapeutic applications and nanogel design studies. Nanogels have a key role to play in the management of ophthalmic disorders, nasal medicine transport, and vaginal drug administration, according to a new study in nanogels and nanotechnology [71]. The booming pharmaceutical business now has a multibillion-dollar market for nanogels produced with natural medications. However, there are still considerable obstacles in the way of using natural medicines in clinical studies. Nanogels appear to have a bright future in biomedical applications, according to recent research [111]. For example, to control diabetes, a poly(4-vinyl phenyl boronic acid-co-2-(dimethylamino) ethyl acrylate) nanogel with insulin-loaded silver nanoparticles has already been developed [112]. According to a report published by the World Health Organization, herbal-based pharmaceuticals will be used by 80% of the world’s population to address their health needs. Despite allopathic pharmaceuticals’ market potential, people seek alternative medicine as a complementary medical practice. Due to considerable changes in people’s attitudes, be it social, political, or economic, the therapeutic application of herbal drugs has dramatically decreased. A viable platform for improving herbal characteristics is nanogel formulations. Natural products are transformed into the most effective pharmaceuticals for treating a variety of ailments, which includes cancer, skin diseases, diabetes, and others, using herbal nanogels. Cross-linked herbal nanogels are commonly made with chitin, chitosan, PLGA, PEG, and other polymers. These cross-linked nanogels show a lot of promise for delivering medications through the skin. This has less adverse effects on patients’ compliance with herbal treatments than oral pharmaceuticals. Despite the fact that numerous natural therapeutic remedies have been created, not all of them are safe. Some are extremely hazardous and can interact with other medications.

## 12. Conclusions

As a result of the advancement of nanotechnology in recent decades, nanocarriers have evolved and gained significance in biomedicine. Nanomedicine is a crucial tool in the fight against novel coronaviruses, but it still confronts substantial obstacles in clinical practice, including in vivo behavior, nanocarrier toxicity, and industrial scale production [113]. Nanocarriers are used as carriers of traditional chemotherapeutic medications and platforms for combinational therapy, multifunctional diagnostics, and theranostics due to their drug entrapment potential. Nanocarriers have been used for passive targeting via the EPR effect, active targeting via ligand modification of nanoplatforms surfaces, and site-specific and time-controlled drug delivery strategies using stimuli-responsive nanocarriers [24]. Nanogels have proven to be superior in terms of reducing the complexity of this delivery system while also eliminating the disadvantages of previous techniques [114]. The immense promise of functional nanogels as unique polymeric platforms for biomedicine is highlighted by applications in the fields of drug and gene delivery, smart imaging modalities, responsive materials, and multivalency as a therapeutic strategy [115].

## Figures and Tables

**Table 1:** Delivery of nanogel incorporated drugs.

Drug	Purpose	Comments	Reference
Pilocarpine	Improve stability and bioavailability	Long and sustained release of pilocarpine	[14]
Fluconazole	Improve corneal bioavailability	The prepared Flu-CNGs showed controlled release of fluconazole	[15]
Timolol meleate	As contact lens with lysozyme triggered release of drug	Controlled and sustained drug release attained	[16]

**Table 2:** Representative examples of some bioactive loaded nanogels.

Nanogel/Herb	Condition	Reference
*Eupatorium adenophorum*	Antibacterial	[93]
*Cleodendron infortunatum*	Bronchitis, asthma, fever, skin ailments, and epilepsy	[94]
Sesbania grandiflora	Anti-acne, anti-peroxidase, analgesic, reducing fever, anti-bacterial, anticancer, thrombolytic, and hepato-immune response	[96]
*Withania somnifera*	Anti-inflammatory	[99]
Albizia lebbeck	Anti-inflammatory and analgesic	[94,95]
Tridax procumbens	Anti-bacterial	[101]
Akkalkara.	Pain reliever	[102]
Mannose	To boost fibroblast activity and collagen production	[103]
Aloe barbadensis	Wound reduction	[103]
Cynodon dactylon Pers. and Cassia tora linn. Cassia alata Linn	Anti-cancer	[104]
